# Machine Learning in Maternity: Early Diagnosis of Placenta Accreta Spectrum

**DOI:** 10.7759/cureus.106592

**Published:** 2026-04-07

**Authors:** Daniel Waszczuk, Varsha Manikandan, Brendan L Wong, Frank Martin, Nitish Bhargava, Annika Mondal, Morgan Loy, Drake Strnad, Manikandan Panchatcharam, Gayathri Sadanala, Sumitra Miriyala

**Affiliations:** 1 Anatomy, A.T. Still University, Kirksville College of Osteopathic Medicine, Kirksville, USA; 2 High School, Kirksville Senior High School, Kirksville, USA; 3 High School, Dulles High School, Sugar Land, USA; 4 Neurology, Saint Louis University School of Medicine, Saint Louis, USA; 5 Computer Science, Truman State University, Kirksville, USA

**Keywords:** diagnostic, machine learning, maternal health, mortality, mri (magnetic resonance imaging), placenta accreta, placenta accreta spectrum, ultrasound imaging

## Abstract

Placenta accreta spectrum (PAS) is a life-threatening obstetric condition marked by abnormal placental attachment and invasion into the uterine wall. Early and accurate prenatal diagnosis is crucial to improving maternal and fetal outcomes as it reduces morbidity, mortality, and complications such as severe hemorrhage and cesarean hysterectomy. Current diagnostic approaches utilize magnetic resonance and ultrasound imaging and rely heavily on clinician interpretation.

This study aims to evaluate recent advancements in machine learning (ML) techniques for the early diagnosis of PAS using ultrasound and magnetic resonance imaging (MRI).

A comprehensive review of the literature evaluated 14 studies highlighting various ML techniques, including linear, ensemble, deep learning, and hybrid models, highlighting the study outcomes.

ML techniques demonstrated improved diagnostic performance compared to traditional methods. Ultrasound-based models achieved accuracy rates ranging from 84.6% to 92.3%, particularly with ensemble methods and deep dictionary learning. MRI-based approaches showed even higher performance, particularly with texture analysis using k-nearest neighbors (k-NN), achieving up to 98.1% accuracy. Despite these promising results, notable challenges included model generalizability across diverse populations and variability in imaging quality due to differences in medical equipment and patient demographics.

Overall, ML offers significant potential as a tool for the early diagnosis of PAS by improving diagnostic accuracy, consistency, and reducing human error. However, further research is needed to address limitations related to generalizability and standardization before widespread clinical implementation.

## Introduction and background

Placenta accreta spectrum (PAS) is a serious and increasingly prevalent obstetric condition in which the placenta abnormally adheres to or invades the uterine wall [1]. Affecting approximately one in 588 pregnancies [2], PAS poses a significant risk of life-threatening hemorrhage during delivery, primarily due to the placenta’s failure to detach from the uterine wall [3]. Cesarean hysterectomy remains the standard intervention to control hemorrhage and reduce complications such as massive blood loss and urinary tract injury [3]. Despite advancements in surgical techniques, patients with PAS remain at high risk for serious surgical and postoperative complications [1,4].

Timely and accurate prenatal diagnosis is essential for optimizing maternal and fetal outcomes [5]. Imaging modalities, particularly ultrasound and magnetic resonance imaging (MRI), are central to the diagnosis and preoperative planning of PAS [6]. However, their diagnostic accuracy is often limited by inter-observer variability and a strong dependence on clinician expertise [7]. To mitigate these limitations, machine learning (ML) offers a promising solution by enabling earlier and more consistent PAS detection through automated image analysis [8,9].

This review synthesizes recent research on ML-based PAS detection, comparing model performance across imaging types and identifying key challenges and gaps in current methodologies. Through critical evaluation of existing evidence, this review aims to clarify the current landscape and propose future directions for integrating ML into routine obstetric imaging workflows.

## Review

Objective

**Figure 1:**
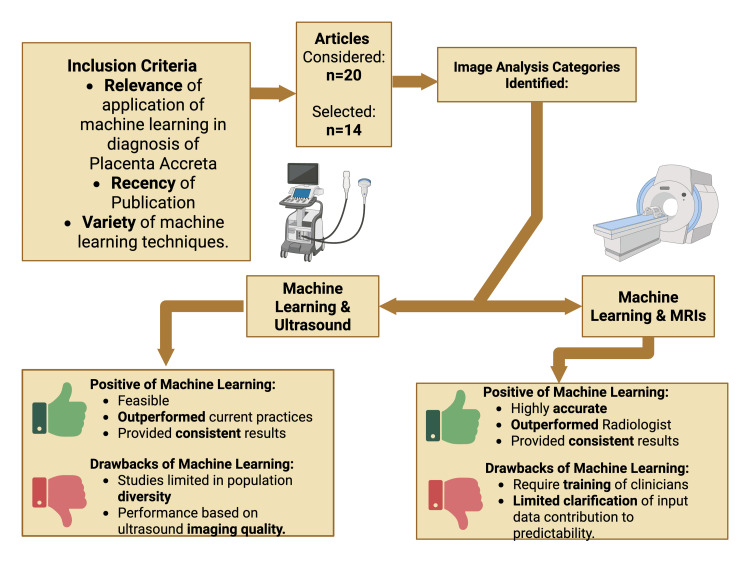
Machine learning in diagnosis of placenta accreta spectrum: a comprehensive review. The figure was created by the author using BioRender (Toronto, Canada).

This review evaluates recent advancements in ML for the prenatal diagnosis of PAS. It synthesizes findings from studies using ML techniques on ultrasound and MRI data to assess improvements in diagnostic accuracy and consistency. The review also explores the clinical potential of ML integration into diagnostic workflows and highlights key challenges, such as data variability and model interpretability, that must be addressed to support broader clinical adoption. See Figure 1 for a graphical overview of the study.

Materials and methods

A narrative review was conducted to identify studies evaluating the application of ML in the prenatal diagnosis of PAS. A comprehensive search was performed using PubMed/MEDLINE and Scopus databases, accessed through the Texas Medical Center Library. The search covered studies published between January 2014 and January 2025 and was limited to articles published in English.

Search terms included combinations of “placenta accreta”, “placenta accreta spectrum”, “machine learning”, “deep learning”, “ultrasound”, “MRI”, and “diagnostic imaging”, using Boolean operators to refine the search strategy.

The literature screening process was conducted independently by two reviewers. Methodological rigor was assessed based on study design, sample size, transparency of ML methodology, and completeness of reported outcomes. Discrepancies between reviewers were resolved through discussion and consensus.

The initial database search identified 91 records. Prior to screening, 10 records were removed using automation tools due to ineligibility, leaving 81 records for title and abstract screening. Of these, 61 records were excluded for not focusing on the application of ML in the diagnosis of PAS.

A total of 20 full-text articles were assessed for eligibility based on predefined inclusion criteria: (1) direct application of ML techniques to PAS diagnosis; (2) use of prenatal imaging data (ultrasound or MRI); (3) reporting of performance metrics such as accuracy, sensitivity, or specificity; (4) publication within the past 10 years; and (5) clear description of ML methodology and clinical context.

**Figure 2:**
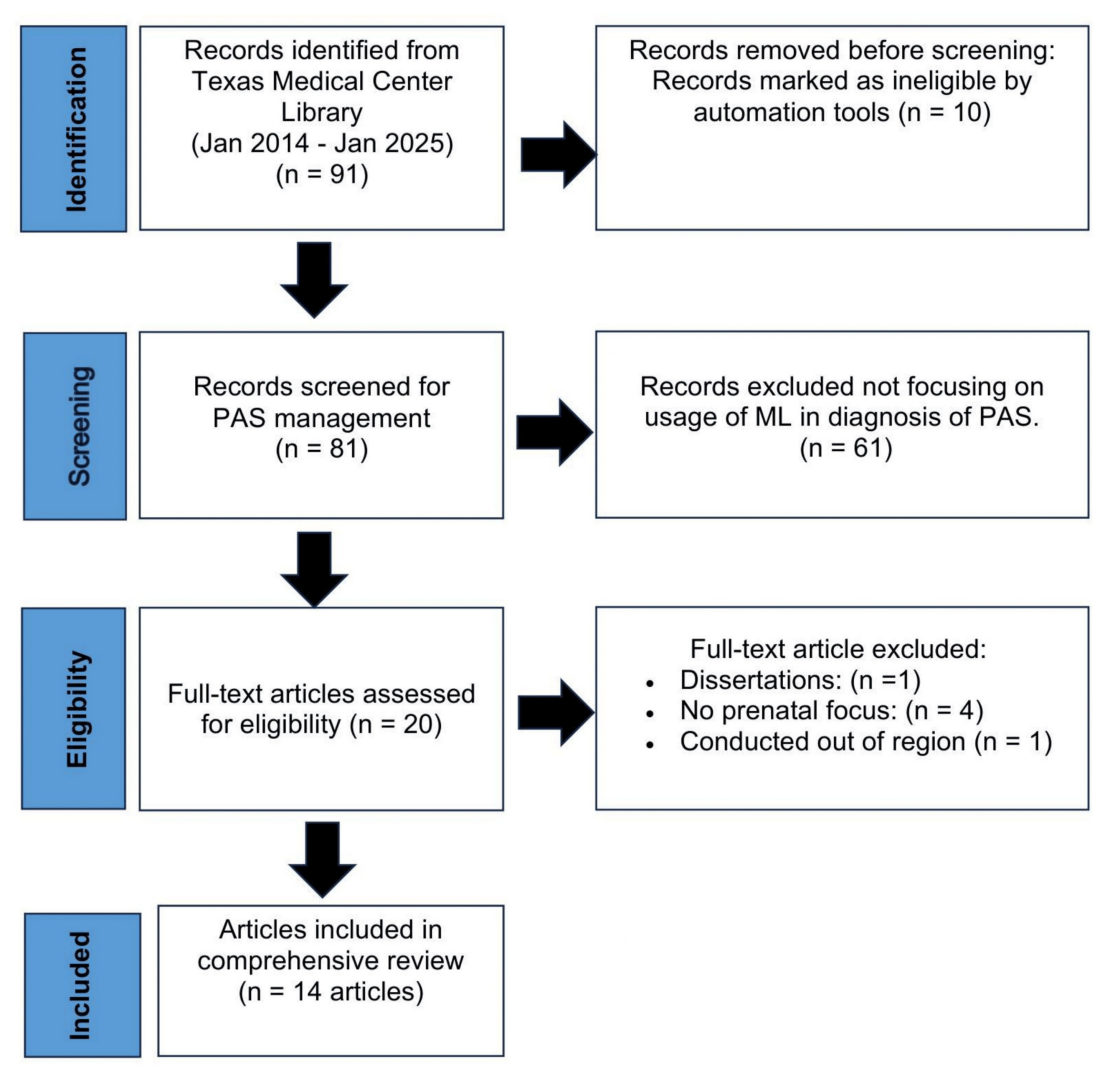
PRISMA flow diagram. PRISMA: Preferred Reporting Items for Systematic Reviews and Meta-Analyses; PAS: placenta accreta spectrum; ML: machine learning.

Of these, six articles were excluded for the following reasons: dissertation format (n = 1), lack of prenatal diagnostic focus (n = 4), and studies conducted outside the defined region (n = 1). Ultimately, 14 studies met all inclusion criteria and were included in the final analysis. The study selection process is summarized in the Preferred Reporting Items for Systematic Reviews and Meta-Analyses (PRISMA) flow diagram (Figure 2).

To enhance clinical relevance, informal expert input was obtained from clinicians at Texas Children’s Pavilion for Women. This input was used solely to contextualize findings and interpret their applicability to current clinical practice. This did not influence study selection or inclusion decisions.

Results

Accurate prenatal diagnosis of PAS remains a global challenge due to human error, limited specialist access, and variability in imaging interpretation, which can lead to delayed or inappropriate clinical interventions [10,11]. In a 2015 observational study in Sweden and Norway, researchers reported that 70% of PAS cases were not diagnosed antepartum. Similarly, a 2014 study in Utah, United States, discovered that up to one-third of cases are not diagnosed during antepartum [12,13].

PAS is traditionally diagnosed prenatally using imaging modalities such as ultrasound and MRI, with ultrasound being the first-line diagnostic tool due to its wide availability, non-invasiveness, and cost-effectiveness [3,7]. MRI is typically used when ultrasound results are inconclusive, offering superior soft tissue contrast and clearer visualization of placental invasion depth and extent [3]. While both modalities contribute significantly to prenatal detection, their accuracy is highly dependent on operator expertise, image quality, and gestational age, which can lead to variability in diagnostic performance and occasional false positives or negatives.

While the advancement of manual diagnosis through the Placenta Accreta Index (PAI) process does improve the accuracy of diagnosis, it does not improve upon the PAS diagnostic issues of manual time consumption, proper symptom identification, and other complexities introduced via human error [10]; however, with ML, these errors can be mitigated, as ML algorithms can be trained to analyze datasets of ultrasound images and MRIs to more accurately identify patterns and symptoms that have high correlation to PAS. ML allows physicians to diagnose PAS swiftly with higher accuracy, potentially improving patient outcomes and mitigating the likelihood of complications associated with delayed or inaccurate diagnosis [11].

Current ML programs do face limitations that require thorough examination. The efficacy of ML algorithms can vary across different populations, as disparities in patient demographics and health backgrounds may affect the program's performance and generalizability [14]. ML programs must contend with varying image quality across different healthcare settings; low-quality images due to inadequate equipment or operator expertise can lead to reduced diagnostic accuracy [12,15]. Also, when diagnosing PAS, ML methods use both ultrasound imaging and MRIs, each with distinct advantages and limitations. A 2019 study identified ultrasound as the first-line imaging modality due to its wide availability and lower cost. However, it remains highly operator-dependent and demonstrates limited reproducibility [16]. In contrast, MRIs are relatively operator independent, but they have a higher cost and can be discomforting to patients [16]. Worldwide, the incidence of PAS is rising with the rising rate of cesarean delivery, and early detection is a pressing global public health concern. Research calculations reveal that undiagnosed PAS is responsible for serious maternal morbidity, particularly in the resource-poor environment where imaging facilities and senior doctors are not readily available. Even in developed countries, late diagnosis often results in emergency interventions that are associated with massive transfusion of blood, hysterectomy, and intensive care admission. These underline the need for novel diagnostic tests that are reproducible, precise, and flexible across a range of clinical environments. ML, with its ability to read complex imaging data [17] and to reduce the dependence on operator skill, has great potential as a method of improving consistency and enabling earlier diagnosis of PAS [6].

Image Analysis Through Machine Learning and Ultrasound

Multiple studies have explored the feasibility of applying ML to ultrasound datasets for PAS diagnosis, using varied training approaches [18,19]. In a study by Young et al. [20], linear and ensemble classifiers were compared for PAS diagnosis using ultrasound texture features [20].

Linear classification, while straightforward, showed limitations in handling complex, non-linear imaging data. In contrast, ensemble classification, combining multiple models, demonstrated improved accuracy and robustness [21,22]. In a shared dataset, both ML methods outperformed manual diagnosis, with ensemble classification achieving the highest accuracy [20]. Ensemble classification achieved 92.3% accuracy, compared to 87.2% for linear models and 84.6% for manual diagnosis [20].

Yang et al. [23] developed a hybrid model combining dictionary learning and deep learning to enhance PAS detection. The model leveraged deep neural networks and matrix decomposition to extract complex imaging features. The positive predictive value (PPV), negative predictive value (NPV), and false positive rate (FPR) of ultrasound image information based on the deep dictionary learning algorithm were 95.33%, 94.89%, and 3.56%, respectively [23]. These results suggest that deep dictionary learning offers a viable and more accurate alternative to manual ultrasound diagnosis, which typically yields PPVs between 82% and 93% [23].

The integration of deep learning techniques consistently outperformed manual diagnostic methods when trained on quality datasets [24-26]. Despite these promising results, ML programs still face the challenges of diagnosing PAS in diverse populations. The highlighted studies [20,21,23] focused on specific demographic groups, limiting the generalizability of their results to broader or underrepresented populations. Image quality variability, particularly in low-resource settings, may further limit ML performance and generalizability. This disparity in performance emphasizes the need for future research to address variations in population demographics and imaging technology quality to ensure the global applicability of ML programs in PAS diagnosis.

Image Analysis Through Machine Learning and MRI

While ultrasound is the first-line modality for PAS diagnosis, MRI is often used when ultrasound results are inconclusive. Several studies have investigated ML-based approaches using MRI for PAS diagnosis [27,28]. Romeo et al. [16] analyzed texture features from T2-weighted MRI using the Pyradiomics platform to extract radiomic features. The study applied multiple ML algorithms and data balancing techniques, such as SMOTE, though it noted a risk of overfitting [16]. The k-nearest neighbor (k-NN) ML algorithm showed the highest accuracy of PAS diagnosis (98.1%) with the fewest features, while naïve Bayes had the lowest (80.5%), indicating variability in ML technique performance [16]. The k-NN model outperformed manual MRI interpretation, which had an accuracy of 92.3% [29].

A 2022 multicenter study by Ye et al. [30] evaluated three ML models for the diagnosis of PAS using MRI data: a radiomics model, a deep learning model, and a clinical model. The radiomics model utilized a support vector machine (SVM) to analyze 860 extracted imaging features. The deep learning model employed a multi-layer neural network that generated predictions based on learned feature weights. In contrast, the clinical model relied solely on preoperative clinical variables and did not incorporate MRI data. Overall, the ensemble approach demonstrated strong classification performance and consistent stability across multiple centers [30].

In a 2024 retrospective study, Wang et al. [31] compared a deep learning model to radiologist performance in diagnosing PAS. They discovered that the ML program outperformed the radiologists, with an area under the curve (AUC) of 0.860 vs. 0.737/0.749/0.770, respectively. The study concluded that ML provided more consistent diagnostic accuracy, reducing variability caused by differing levels of radiological expertise. Wang et al. [31] concluded that their program could normalize the varying levels of manual diagnostic PAS expertise through the minimization of PAS diagnosis to their one ML program. A study by Li et al. [32] developed an ML model using the CatBoost algorithm to predict adverse clinical outcomes in PAS, achieving AUCs of 0.90 (internal) and 0.84 (external), with implementation as an online prediction tool.

Overall, ML models demonstrate improved diagnostic performance and consistency compared to manual MRI interpretation.

Recent Advancements in PAS Diagnosis via Machine Learning

Over the last five years, the application of ML in diagnosing PAS has undergone significant evolution, with researchers exploring more complex neural networks, improved feature extraction techniques, and larger, multi-institutional datasets to enhance diagnostic performance [33-35].

For example, Young et al. demonstrated that ensemble classifiers significantly outperformed linear models and manual interpretation in ultrasound-based PAS diagnosis [20]. The ensemble model achieved 92.3% accuracy, reinforcing the value of model fusion in improving diagnostic reliability [20]. In perspective, another important contribution came from Yang et al., who proposed a hybrid deep dictionary learning model that merged deep learning with matrix decomposition techniques. Their model was trained on clinical ultrasound datasets and achieved a PPV of 95.33%, demonstrating a substantial improvement over traditional PPVs of 82-93% [23]. The study highlighted how advanced model design can improve sensitivity and specificity, particularly in variable-quality ultrasound data.

On the MRI front, Ye et al. conducted a multicenter study that integrated three ML models, radiomics, deep learning, and clinical data, into a combined prediction system. Using a dataset of T2-weighted MRI, the combined model achieved high stability and diagnostic accuracy across different clinical centers, reinforcing the strength of multimodal ML integration [30]. In comparison, Wang et al. evaluated a deep learning pipeline trained on retrospective MRI data, showing that their model surpassed radiologists in diagnostic accuracy with an AUC of 0.860 compared to 0.737-0.770 for radiologist interpretation. Both of these studies demonstrated the potential for ML systems to augment, and in some cases outperform, traditional diagnostic expertise, especially in settings with limited radiological consistency [31].

More recent studies have further advanced ML-based PAS diagnosis through improved model architecture and clinical applicability. Danaei et al. emphasized the practical applications of ML in PAS by applying convolutional neural networks (CNNs) to ultrasound data, further supporting the real-world feasibility of automated diagnosis in high-volume obstetric settings. By leveraging a curated dataset from multiple tertiary care centers, the model maintained robust performance across varied patient demographics and imaging protocols. These findings support ML’s utility in real-world settings where diagnostic consistency is often challenged by operator variability [36]. Comparatively, Hu et al. evaluated the DenseNet-121 model, a deep CNN across multiple centers. The model not only enhanced diagnostic accuracy but also improved interobserver reliability among radiologists. Notably, Hu et al. incorporated saliency mapping techniques, which visually highlighted the most predictive regions on MRI scans. This interpretability aspect is crucial for clinical trust and adoption, as it allows physicians to understand and validate the decision-making process of the ML model [37].

Collectively, these findings support the reliability of ML models in outperforming manual MRI interpretation and reducing diagnostic inconsistencies. A summary of ML models is shown in Table 1.

**Table 1:** Comparative summary of machine learning models for PAS diagnosis. ML: machine learning; PPV: positive predictive value; NPV: negative predictive value; CNN: convolutional neural network; k-NN: k-nearest neighbor; MRI: magnetic resonance imaging; AUC: area under the curve; DL: deep learning; SHAP: SHapley Additive exPlanations; FIGO: International Federation of Gynecology and Obstetrics; PAS: placenta accreta spectrum; DPMM: Dirichlet process mixture models.

Study	Modality	ML technique	Dataset size/Source	Performance metrics	Interpretability
Young et al. (2024) [20]	Ultrasound	Ensemble classifier	300 cases, single center	Accuracy: 92.3%	None
Yang et al. (2022) [23]	Ultrasound	Deep dictionary learning	250 cases	PPV: 95.3%, NPV: 94.9%	Not specified
Danaei et al. (2025) [36]	Ultrasound	CNN (nnU-Net)	Multi-institutional	AUC: High	Not reported
Danaei et al. (2025) [36]	MRI	DenseNet-PAS	Not specified	AUC: High	Not reported
Hu et al. (2025) [37]	MRI	DenseNet-121 + Saliency maps	263 cases, 2 centers	AUC: 0.956 (train), 0.863 (external)	Saliency mapping
Romeo et al. (2019) [16]	MRI	k-NN	120 cases	Accuracy: 98.1%	None
Ye et al. (2022) [30]	MRI	Ensemble (Radiomics + DL + Clinical)	3 centers	AUC: High/stable	Calibration curves
Wang et al. (2024) [31]	MRI	Deep learning pipeline	200 retrospective cases	AUC: 0.860	None
Zou et al. (2025) [24]	MRI	Radiomics + Clinical	2-center cohort	AUC: 0.918 (train), 0.885 (test)	Feature importance
Zou et al. (2025) [24]	MRI	Radiomics + Nomogram	3 centers	AUC: High	Nomogram
Jiang et al. (2025) [19]	MRI	Multitask CNN	4,140 MRI slices	Accuracy: High	Anatomical attention
Bartels et al. (2023) [25]	MRI	Radiomics	100+ cases	FIGO grade prediction	Not reported
Guo et al. (2025) [33]	MRI	Radiomics + Clinical	3 centers	AUC: 0.89	SHAP
Li et al. (2025) [32]	MRI + clinical	CatBoost + SHAP	125 cases, 2 centers	AUC: 0.90 (internal), 0.84 (external)	SHAP

These recent studies reflect a rapid shift toward using ensemble methods, deep learning architectures, and hybrid pipelines trained on larger, more diverse datasets. This growing body of work shows that ML models are not only feasible for clinical use but may eventually standardize and streamline PAS diagnosis across institutions. As ML adoption expands, future efforts should focus on improving model robustness to image quality variability and addressing clinician trust and usability concerns.

Challenges and limitations

While ML holds promise for improving PAS diagnosis, several challenges hinder its clinical integration, particularly those related to data quality, generalizability, and interpretability. The availability of high-quality and annotated datasets is essential for training robust ML programs, but access to these datasets is often restricted due to patient privacy and security concerns [38]. Imaging modalities continue to evolve, leading to variability in dataset characteristics over time [39]. These changes necessitate adaptable ML models capable of learning from evolving imaging standards. Bias in data also presents a critical issue, as ML algorithms that have been trained on biased datasets may show disparities in performance across different population groups, leading to decreased diagnostic accuracy in specific demographic groups [40]. Effective implementation also depends on clinician training, as many ML tools require a foundational understanding of algorithmic outputs and limitations [38]. These challenges are particularly relevant in PAS diagnosis, where imaging variability and clinical urgency demand high model reliability [33,41].

**Figure 3:**
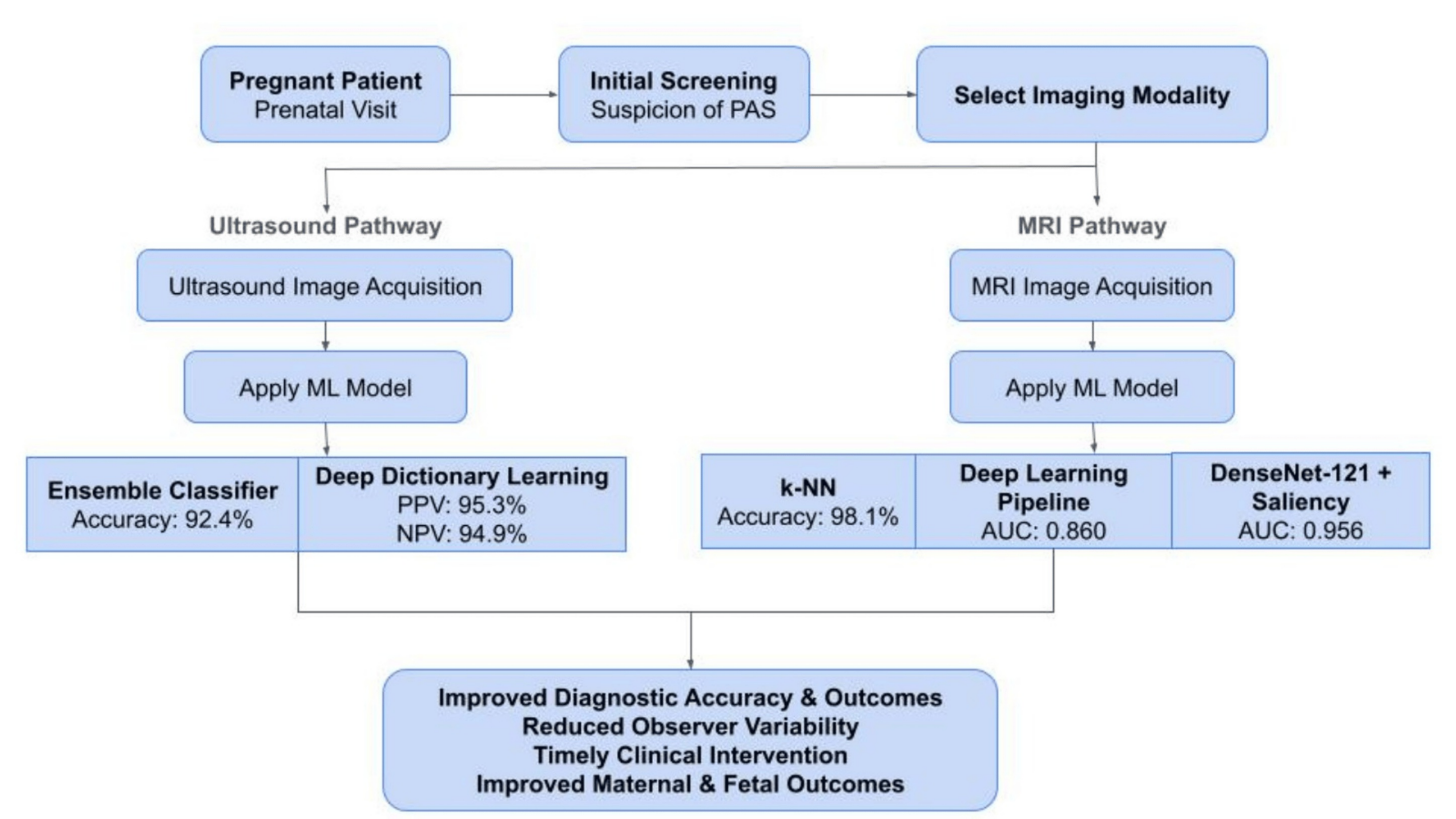
Machine learning-enhanced diagnostic workflow for placenta accreta spectrum. ML: machine learning; PPV: positive predictive value; NPV: negative predictive value; k-NN: k-nearest neighbor; MRI: magnetic resonance imaging; AUC: area under the curve; PAS: placenta accreta spectrum.

A key barrier to adoption is clinician trust, as many remain cautious about relying on opaque ML decision-making processes [42]. ML program integration runs the risk of overfitting and brittleness, meaning that ML may perform accurately given pre-diagnosed training data, but ML may eventually struggle with new or undiagnosed inputs. Such diagnostic unreliability may undermine confidence in ML tools, limiting their adoption despite demonstrated performance benefits [39]. The ML workflow for PAS detection is shown in Figure 3.

Discussion and future research

Continued research into ML-based diagnostic tools offers significant potential to improve the accuracy and efficiency of PAS detection. Future studies should explore ML models that integrate multimodal data, such as imaging, clinical history, and laboratory results, to address issues like overfitting and brittleness. For instance, combining electronic health records (EHRs), genetic markers, and imaging data could enhance the predictive accuracy of ML programs by providing a more comprehensive view of the patient’s health profile. Additionally, methods for mitigating bias in ML programs should be explored to ensure consistent performance across diverse demographic groups by using more representative datasets. This includes curating datasets that reflect diverse ethnic and socioeconomic backgrounds to reduce health disparities. The reliability of these diagnostic tools could also be improved by looking into specific methods dealing with variable datasets, such as continual learning algorithms that adjust to changing data distributions over time. Another focus of further studies might be on enhancing program interpretability, making the ML program’s decision-making process more transparent and trustworthy for clinicians. Techniques like attention mechanisms or saliency maps can help visualize which parts of the input data (ultrasound and MRI scans) contribute the most to the program’s predictions, further allowing clinicians to understand and grow confidence in the outputs. Addressing these areas will be critical for advancing ML integration into PAS diagnosis and improving early detection and clinical outcomes.

Looking ahead, ML models such as DenseNet-121 and k-NN have the potential to augment traditional diagnostic approaches, particularly in resource-limited settings. As these models continue to demonstrate improved diagnostic accuracy and consistency, they may serve as valuable decision support tools alongside standard clinical assessment. Integration of ML into imaging workflows could enhance real-time interpretation and reduce variability associated with operator expertise. However, further validation across diverse populations and clinical settings is necessary before widespread implementation. With continued improvements in interpretability, scalability, and generalizability, ML models are well-positioned to support clinicians in achieving more accurate and equitable detection of PAS.

## Conclusions

Applying ML to standard imaging techniques such as ultrasound and MRI has demonstrated potential to enhance the prenatal diagnosis of PAS. As demonstrated by the studies reviewed, ML-based approaches, including linear, ensemble, deep learning, and hybrid models, showed improved diagnostic performance and reduced variability compared to traditional manual interpretation alone. Ultrasound-based models achieved strong accuracy, which is particularly intriguing given the availability of the imaging modality. MRI, on the other hand, while less readily available, achieved the highest performance in several studies. These findings highlight the growing capability of ML to assist in identifying complex imaging patterns associated with PAS.

However, despite these promising results, several limitations remain. Variability in imaging quality, differences in patient populations, and inconsistencies in study design continue to affect the generalizability of ML models across diverse clinical settings. Additionally, reliance on retrospective datasets and variability in reported performance metrics may limit direct comparison between studies. These gaps underscore the need for future research focusing on larger, more diverse datasets and standardized evaluation methods to improve model reliability and comparability. Overall, ML represents a valuable adjunct to current diagnostic approaches, with the potential to improve diagnostic consistency and support clinical decision-making in PAS, particularly in settings where expertise may be limited.

## References

[1] Fonseca A, Ayres de Campos D (2021). Maternal morbidity and mortality due to placenta accreta spectrum disorders. Best Pract Res Clin Obstet Gynaecol.

[2] Jauniaux E, Hussein AM, Fox KA, Collins SL (2019). New evidence-based diagnostic and management strategies for placenta accreta spectrum disorders. Best Pract Res Clin Obstet Gynaecol.

[3] Einerson BD, Gilner JB, Zuckerwise LC (2023). Placenta accreta spectrum. Obstet Gynecol.

[4] Rac MW, Dashe JS, Wells CE, Moschos E, McIntire DD, Twickler DM (2015). Ultrasound predictors of placental invasion: the Placenta Accreta Index. Am J Obstet Gynecol.

[5] Saida T, Gu W, Hoshiai S (2025). Artificial intelligence in obstetric and gynecological MR imaging. Magn Reson Med Sci.

[6] Cahill AG, Beigi R, Heine RP, Silver RM, Wax JR (2018). Placenta accreta spectrum. Am J Obstet Gynecol.

[7] Horgan R, Abuhamad A (2022). Placenta accreta spectrum: prenatal diagnosis and management. Obstet Gynecol Clin North Am.

[8] Arain Z, Iliodromiti S, Slabaugh G, David AL, Chowdhury TT (2023). Machine learning and disease prediction in obstetrics. Curr Res Physiol.

[9] Ramirez Zegarra R, Ghi T (2023). Use of artificial intelligence and deep learning in fetal ultrasound imaging. Ultrasound Obstet Gynecol.

[10] Ahsan MM, Luna SA, Siddique Z (2022). Machine-learning-based disease diagnosis: a comprehensive review. Healthcare (Basel).

[11] Habehh H, Gohel S (2021). Machine learning in healthcare. Curr Genomics.

[12] Thurn L, Lindqvist PG, Jakobsson M (2016). Abnormally invasive placenta—prevalence, risk factors and antenatal suspicion: results from a large population-based pregnancy cohort study in the Nordic countries. BJOG.

[13] Bowman ZS, Eller AG, Bardsley TR, Greene T, Varner MW, Silver RM (2014). Risk factors for placenta accreta: a large prospective cohort. Am J Perinatol.

[14] Moannaei M, Jadidian F, Doustmohammadi T (2025). Performance and limitation of machine learning algorithms for diabetic retinopathy screening and its application in health management: a meta-analysis. Biomed Eng Online.

[15] Shen D, Wu G, Suk HI (2017). Deep learning in medical image analysis. Annu Rev Biomed Eng.

[16] Romeo V, Ricciardi C, Cuocolo R (2019). Machine learning analysis of MRI-derived texture features to predict placenta accreta spectrum in patients with placenta previa. Magn Reson Imaging.

[17] Laçi H, Sevrani K, Iqbal S (2025). Deep learning approaches for classification tasks in medical X-ray, MRI, and ultrasound images: a scoping review. BMC Med Imaging.

[18] Singh R, Gupta S, Mohamed HG, Bharany S, Rehman AU, Ghadi YY, Hussen S (2025). Advancing prenatal healthcare by explainable AI enhanced fetal ultrasound image segmentation using U-Net++ with attention mechanisms. Sci Rep.

[19] Jiang H, Liu Q, Zhou Y, Pan J, Song T, Lu Y (2025). Anatomy-guided multitask learning for MRI-based classification of placenta accreta spectrum and its subtypes. arXiv.

[20] Young D, Khan N, Hobson SR, Sussman D (2024). Diagnosis of placenta accreta spectrum using ultrasound texture feature fusion and machine learning. Comput Biol Med.

[21] Brattain LJ, Telfer BA, Dhyani M, Grajo JR, Samir AE (2018). Machine learning for medical ultrasound: status, methods, and future opportunities. Abdom Radiol (NY).

[22] Cross JL, Choma MA, Onofrey JA (2024). Bias in medical AI: implications for clinical decision-making. PLOS Digit Health.

[23] Yang X, Chen Z, Jia X (2022). Deep learning algorithm-based ultrasound image information in diagnosis and treatment of pernicious placenta previa. Comput Math Methods Med.

[24] Zou J, Wei W, Xiao Y, Wang X, Wang K, Xie L, Liang Y (2025). Predicting placenta accreta spectrum and high postpartum hemorrhage risk using radiomics from T2-weighted MRI. BMC Pregnancy Childbirth.

[25] Bartels HC, O'Doherty J, Wolsztynski E (2023). Radiomics-based prediction of FIGO grade for placenta accreta spectrum. Eur Radiol Exp.

[26] Zhang Y, Ellestad SC, Gilner JB, Pyne A, Boyd BK, Mazurowski MA, Gatta LA (2024). Pilot study of machine learning for detection of placenta accreta spectrum. Ultrasound Obstet Gynecol.

[27] Yaşar Ş, Yoloğlu S (2022). Prediction of placenta accreta spectrum by machine learning methods and determination of candidate biomarkers. J Cogn Syst.

[28] Kothinti RR (2024). Enhancing prenatal diagnosis: AI-driven multimodal prediction of fetal brain and heart abnormalities using ultrasound and MRI data. J Emerg Technol Innov Res.

[29] Fiocchi F, Monelli F, Besutti G (2020). MRI of placenta accreta: diagnostic accuracy and impact of interventional radiology on foetal-maternal delivery outcomes in high-risk women. Br J Radiol.

[30] Ye Z, Xuan R, Ouyang M, Wang Y, Xu J, Jin W (2022). Prediction of placenta accreta spectrum by combining deep learning and radiomics using T2WI: a multicenter study. Abdom Radiol (NY).

[31] Wang H, Wang Y, Zhang H (2024). A deep learning pipeline using prior knowledge for automatic evaluation of placenta accreta spectrum disorders with MRI. J Magn Reson Imaging.

[32] Li H, Zhang Y, Mei H (2025). Development and validation of an interpretable machine learning model for predicting adverse clinical outcomes in placenta accreta spectrum: a multicenter study. Acad Radiol.

[33] Guo C, Guo S, He C (2025). Comparisons among radiologist, MR findings and radiomics-clinical models in predicting placenta accreta spectrum disorders: a multicenter study. Arch Gynecol Obstet.

[34] Song K, Feng J, Chen D (2024). A survey on deep learning in medical ultrasound imaging. Front Phys.

[35] Shobayo O, Saatchi R (2025). Developments in deep learning artificial neural network techniques for medical image analysis and interpretation. Diagnostics (Basel).

[36] Danaei M, Yeganegi M, Azizi S (2025). Machine learning applications in placenta accreta spectrum disorders. Eur J Obstet Gynecol Reprod Biol X.

[37] Hu Y, Liu T, Pang S, Ling X, Wang Z, Li W (2025). Deep learning-assisted diagnosis of placenta accreta spectrum using the DenseNet-121 model: a multicenter, retrospective study. J Imaging Inform Med.

[38] Petersson L, Larsson I, Nygren JM (2022). Challenges to implementing artificial intelligence in healthcare: a qualitative interview study with healthcare leaders in Sweden. BMC Health Serv Res.

[39] Kelly CJ, Karthikesalingam A, Suleyman M, Corrado G, King D (2019). Key challenges for delivering clinical impact with artificial intelligence. BMC Med.

[40] Chung CT, Lee S, King E, Liu T, Armoundas AA, Bazoukis G, Tse G (2022). Clinical significance, challenges and limitations in using artificial intelligence for electrocardiography-based diagnosis. Int J Arrhythmia.

[41] Ennab M, Mcheick H (2024). Enhancing interpretability and accuracy of AI models in healthcare: a comprehensive review on challenges and future directions. Front Robot AI.

[42] Hassan M, Kushniruk A, Borycki E (2024). Barriers to and facilitators of artificial intelligence adoption in health care: scoping review. JMIR Hum Factors.

